# Calcium isotopes as a biomarker for vascular calcification in chronic kidney disease

**DOI:** 10.1093/mtomcs/mfad009

**Published:** 2023-02-17

**Authors:** Anthony Dosseto, Kelly Lambert, Hicham I Cheikh Hassan, Andrew Fuller, Addison Borst, Florian Dux, Maureen Lonergan, Theo Tacail

**Affiliations:** Wollongong Isotope Geochronology Laboratory, School of Earth, Atmospheric and Life Sciences, University of Wollongong, Wollongong, New South Wales, Australia; School of Medicine, University of Wollongong, Wollongong, New South Wales, Australia; School of Medicine, University of Wollongong, Wollongong, New South Wales, Australia; Department of Nephrology, Wollongong Hospital, Wollongong, New South Wales, Australia; Wollongong Isotope Geochronology Laboratory, School of Earth, Atmospheric and Life Sciences, University of Wollongong, Wollongong, New South Wales, Australia; School of Medicine, University of Wollongong, Wollongong, New South Wales, Australia; Wollongong Isotope Geochronology Laboratory, School of Earth, Atmospheric and Life Sciences, University of Wollongong, Wollongong, New South Wales, Australia; School of Medicine, University of Wollongong, Wollongong, New South Wales, Australia; Wollongong Isotope Geochronology Laboratory, School of Earth, Atmospheric and Life Sciences, University of Wollongong, Wollongong, New South Wales, Australia; Department of Nephrology, Wollongong Hospital, Wollongong, New South Wales, Australia; Institute of Geosciences, Johannes Gutenberg University, Mainz, Germany

**Keywords:** biomarker, calcium, chronic kidney disease, isotopes, mineral balance, vascular calcification

## Abstract

Calcium balance is abnormal in adults with chronic kidney disease (CKD) and is associated with the development of vascular calcification. It is currently not routine to screen for vascular calcification in CKD patients. In this cross-sectional study, we investigate whether the ratio of naturally occurring calcium (Ca) isotopes, ^44^Ca and ^42^Ca, in serum could be used as a noninvasive marker of vascular calcification in CKD.

We recruited 78 participants from a tertiary hospital renal center: 28 controls, 9 subjects with mild–moderate CKD, 22 undertaking dialysis and 19 who received a kidney transplant. For each participant, systolic blood pressure, ankle brachial index, pulse wave velocity, and estimated glomerular filtration rate were measured, along with serum markers. Calcium concentrations and isotope ratios were measured in urine and serum. While we found no significant association between urine Ca isotope composition (noted δ^44/42^Ca) between the different groups, δ^44/42^Ca values in serum were significantly different between healthy controls, subjects with mild–moderate CKD and those undertaking dialysis (*P* < 0.01). Receiver operative characteristic curve analysis shows that the diagnostic utility of serum δ^44/42^Ca for detecting medial artery calcification is very good (AUC = 0.818, sensitivity 81.8% and specificity 77.3%, *P* < 0.01), and performs better than existing biomarkers. Although our results will need to be verified in prospective studies across different institutions, serum δ^44/42^Ca has the potential to be used as an early screening test for vascular calcification.

## Graphical Abstract

**Graphical Abstract:**
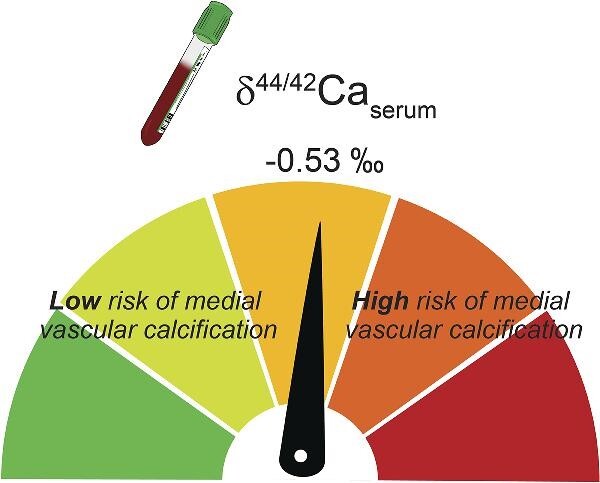
The calcium isotope composition (noted δ^44/42^Ca) of blood serum of adults with chronic kidney disease can be used as a screening tool to detect medial vascular calcification, where a δ^44/42^Ca value greater than −0.53‰ indicates a high risk of medial vascular calcification.

## Introduction

Chronic kidney disease (CKD) is a global health problem affecting almost 10% of the global population.^1^ The largest contributor to morbidity and mortality in patients with CKD is cardiovascular disease, which can affect almost half of patients with severe CKD, in addition to being the leading cause of death in those with kidney failure (KF).^2^ One of the strongest predictors of cardiovascular risk is vascular calcification.^3^ To date no intervention has been shown to be effective in reversing vascular calcification in CKD and only a few have been shown to reduce the progression.^4^ One reason for a lack of therapeutic intervention is the difficulty in treating vascular calcification directly. Current therapies have instead focused on restoring the disrupted mineral balance often seen in CKD such as phosphate control, vitamin D deficiency, vitamin K deficiency, and control of hyperparathyroidism. Another reason why vascular calcification has been a difficult therapeutic target to aim for is the lack of appropriate tools to diagnose vascular calcification in the early stage, when an intervention or lifestyle modification may achieve reversible results. Current tools to diagnose vascular calcification rely exclusively on imaging such as echocardiogram, nuclear medicine scans, or computed tomographic (CT) coronary angiography. These tests are expensive and require exposing the patient to radiation. They are also inappropriate tests to implement as a tool to screen the CKD population due to expense, time needed for each individual test and experienced personnel required to perform and interpret the test. Another limitation is the inability to detect an abnormality until after permanent and irreversible changes have set in (such as the case of CT coronary angiography detecting the presence of permanent vascular calcification).^5^ The ideal screening test for vascular calcification in the CKD population would be easily obtained (such as a blood or urine sample), run in bulk and with results that can be interpreted easily by health professionals without having to be reported by specialists.

Calcium (Ca) has several naturally occurring isotopes, including ^40^Ca, ^42^Ca, ^43^Ca, and ^44^Ca. The distribution of these isotopes in biological fluids and tissues changes in response to biological processes. This distribution is expressed as the ratio of two isotopes, generally ^44^Ca/^42^Ca, thereafter termed Ca isotope ratio and noted δ^44/42^Ca (see “Methods”). Calcium isotopes, as measured in biological fluids such as blood or urine, reflect the global mineral balance of the organism as the result of two combined processes. First, bone is depleted in heavy isotopes (low δ^44/42^Ca), because of a preferential uptake of light Ca isotopes from blood upon bone mineralization (by −0.30‰ or less, e.g. Toepfer *et al*.^6^). Hence, release or uptake of bone Ca tends to, respectively, induce a decrease or increase of serum δ^44/42^Ca values. Second, Ca renal excretion results in the preferential loss of heavy Ca isotopes in urine, higher by +1.2‰ when compared to blood. As a feedback effect, the organism tends to retain light Ca isotopes, δ^44/42^Ca of blood being on average decreased by ca. −0.6‰.^7^,^8^ The increase or decrease of Ca renal excretion (occurring in negative or positive mineral balance, respectively) contributes to a decrease or increase of urine and blood δ^44/42^Ca, respectively.

Overall, on the one hand, negative mineral balance (e.g. induced or pathological bone loss) produces a decrease in serum and urine isotope compositions (e.g. Morgan *et al*., Heuser *et al*., and Eisenhauer *et al*.^9–11^). Morgan *et al*.^9^ for instance proposed that the Ca isotope ratio of urine could be used as a tracer of bone loss, and their bed rest study showed that urine Ca isotope ratios decreased after 1 wk, signaling bone loss long before it could be detected by densitometry. On the other hand, calcification in the body, such as in net bone accretion, is illustrated by an increase in the Ca isotope ratio of urine or blood.^11–13^ Recently, Shroff *et al*.^14^ used Ca isotopes in blood, urine and feces of children with CKD to successfully identify changes in bone Ca balance. While Ca isotopes have been tested and conceptualized in the context of non-ectopic calcification, they are yet to be used for ectopic calcification.

Given the pathological changes occurring in CKD, where bone de-mineralization and disrupted mineral balance lead to an increased risk of vascular calcification, calcium isotopes may be a potential therapeutic test to identify patients at risk of vascular calcification. We therefore set out to examine whether the Ca isotope ratio of urine or blood could be used as an effective biomarker of CKD vascular calcification by conducting an observational cohort study in a population of healthy volunteers, patients with CKD and patients with KF on dialysis. The aims are to (i) assess changes of Ca isotope ratio in urine or blood across CKD stages including dialysis and transplant patients, (ii) examine any association between the Ca isotope ratio of urine or blood, and traditional markers of vascular changes (e.g. FGF-23^15^) and pulse wave velocity (PWV), (iii) evaluate the diagnostic accuracy of urine and serum Ca isotopes for identifying vascular calcification.

## Methods

A total of 78 adult participants were recruited in 2018 from a single renal center (*N* = 28 controls, *N* = 9 with mild–moderate CKD, *N* = 22 with KF undertaking dialysis, *N* = 19 with KF who had received a kidney transplant). Sample size was determined by assessing the previous literature and the study population used to ensure statistical power. A previous study conducted by Channon *et al*.,^16^ recruited 12 healthy individuals (8 males) and found statistical significance between the parameters investigated despite the small sample size. We decided to recruit at minimum the same sample size for each group, with a maximum number of participants at 28, due to funding and time constraints. Recruitment took place at the Wollongong Hospital Renal Unit. We recruited patients with any stage of CKD, patients on dialysis and patients with a functional kidney transplant. Participants were recruited by a renal nurse upon presentation for routine urine analysis at the Wollongong Hospital Renal Unit. Patients who agreed to participate in the study were given study information sheets as well as provided informed consent. Kidney transplant recipients and home hemodialysis patients who do not visit a renal center routinely were sent an invitation to participate. A follow up phone call by an independent research nurse at the renal unit was used to ascertain eligibility and interest in the study. Further amendments to ethics applications (2019/ETH03747) allowed mail out invitations to focus on stage 3 and transplant participants. A convenience sample of healthy controls was recruited. These consisted of staff members or partners/family of staff or participants with CKD. Ethics approval was granted by the Human Research Ethics Committee (Health and Medical) of the University of Wollongong (HREC number: HREC/18/WGONG/188). Inclusion criteria for the study were: (i) individuals suffering any stage of CKD over the age of 18, and (ii) healthy controls that were free from a diagnosis of CKD and were over the age of 18. Exclusion criteria were: (i) adults with a diagnosis of advanced dementia or severe cognitive impairment that would impact informed consent or compliance with study instructions; (ii) individuals with metastatic cancer in the bones to reduce confounding results, as well as reduce added burden to the individual; (iii) individuals with multiple myeloma or other conditions known to impact bone turnover not associated with CKD; and (iv) individuals who were aneuric (<5 mL urine per day).

Urine and blood were collected following overnight fasting. For each participant, age, presence of comorbidities and gender were recorded. Blood pressure, ankle brachial index (ABI), brachial-ankle pulse wave velocity (ba-PWV), and estimated glomerular filtration rate (eGFR) were measured, along with serum albumin, calcium (Ca), phosphate, parathyroid hormone (PTH), alkaline phosphatase level (ALP), 1,25OH vitamin D, creatinine and fibroblast growth factor 23 (FGF23) concentrations. PWV is a noninvasive measurement of arterial stiffness, whereby PWV value increases with arterial stiffness. In CKD transplant and dialysis patients, PWV is positively correlated to cardiovascular mortality.^17^ ABI and ba-PWV measures were conducted using a noninvasive vascular screening device (Omron Colin VP-1000) following the method outlined in Chen *et al*.^18^

In a subset of patients (due to time and funding constraints), Ca concentration and isotope ratio were measured in urine (*N* = 52 for Ca concentration, *N* = 36 for Ca isotope ratio) and serum (*N* = 36 for Ca concentration, *N* = 54 for Ca isotope ratio). For Ca concentration and isotope ratio measurements, urine and serum were freeze dried and then mineralized in nitric acid and hydrogen peroxide by microwave digestion. Samples were then re-dissolved in 2 mol/L HNO_3_ for ion exchange chromatography. An aliquot was taken and diluted in 0.3 mol/L HNO_3_ for measurement of calcium concentrations (see following text).

Ion exchange chromatography was performed to isolate Ca from the sample's matrix, a necessary step for isotope ratio measurement. This was performed using a prepFAST-MC automated chromatography system (ESI, Omaha, NE, USA) at the Wollongong Isotope Geochronology Laboratory (WIGL) following the method outlined in Romaniello *et al*.^19^ The Ca elution was re-dissolved in 0.05 mol/L HNO_3_ for isotope ratio measurement.

Calcium concentration determination was performed by quadrupole inductively coupled plasma mass spectrometry (Q ICP–MS) on a ThermoFisher iCAP-Q at WIGL. A calibration curve was produced using a multi-element standard (Inorganic Ventures 71A) with concentrations ranging from 0.5 to 250 ng/g. An internal standard (Inorganic Ventures 71D) was introduced along with the samples and ^45^Sc was measured to account for instrument drift.

Calcium isotopes were measured on a ThermoFisher Neptune Plus multi-collector ICP–MS at WIGL. A 100 μL/min PFA nebulizer was used with a CETAC Aridus II desolvator as sample introduction system, along with jet sample and X skimmer cones. To account for mass bias, a 1.5 μg/g solution of Alfa Aesar Specpure Ca elemental standard in 0.05 mol/L HNO_3_ was measured before and after each sample following the standard-sample bracketing method.^20^ The Ca concentration of samples in 0.05 mol/L HNO_3_ was adjusted to match that of the primary standard within 10%. Instrument blanks were measured before each standard and sample and subtracted from each isotope. ^42^Ca, ^43^Ca, and ^44^Ca were collected in Faraday cups in medium resolution mode, for 40 cycles of 4.194 s each. Mass bias factors was calculated for ^42^Ca/^43^Ca, ^42^Ca/^43^Ca, and ^43^Ca/^44^Ca ratios. Mass 43.5 was also collected to measure ^87^Sr^2+^, and assess the contribution of ^86^Sr^2+^ and ^88^Sr^2+^ isobaric interferences on ^43^Ca and ^44^Ca, respectively. The 43.5/44 ratio was generally <10^−^^5^, such that no correction for isobaric interference was necessary (the change imparted by the correction on the ^44^Ca/^42^Ca would be within the analytical error). If the 43.5/44 ratio was greater than 10^−^^5^, the analysis was rejected. Furthermore, the analysis was also rejected if either the ^44^Ca intensity of the sample deviated from that of the standard by more than 10%, or the absolute value of the 2sd of the three mass bias factors was greater than 0.1.

The ^44^Ca/^42^Ca and ^43^Ca/^42^Ca isotope ratios were converted to δ^44/42^Ca_WIGL_ and δ^43/42^Ca_WIGL_ values, respectively, expressed in per-mille (‰) and defined as (for instance for δ^44/42^Ca_WIGL_):


(1) \begin{equation*}{\delta ^{\mathrm{44/42}}}{{\mathrm{Ca}}}_{{\mathrm{WIGL}}}{\mathrm{\ = \ }}\left( {{\mathrm{\ }}\frac{{{\mathrm{(^{44}Ca/^{42}Ca)_{sample}}}}}{{{\mathrm{(^{44}Ca/^{42}Ca)_{WIGL}}}}}{\mathrm{ - \ 1\ }}} \right){\mathrm{.\ 1000}}\end{equation*}

where (^44^Ca/^42^Ca)_WIGL_ is the ^44^Ca/^42^Ca ratio of the Alfa Aesar Specpure Ca standard solution. In addition to the filters mentioned earlier, the analysis was rejected if the 2sd of δ^44/^^42^Ca_WIGL _× 0.50667 and δ^43/42^Ca_WIGL_ exceeded 0.1 (indicating deviation for mass-dependent isotope fraction).

The δ^44/42^Ca_WIGL_ values can be converted to δ^44/42^Ca relative to “ICP Ca Lyon,”^8^,^21^,^22^ “ICP1,”^9^,^16^,^23^ or NIST SRM 915a^10^,^24^ standards by adding 0.009‰, 0.277‰, or 0.527‰, respectively. δ^44/42^Ca_WIGL_ values were then converted to δ^44/42^Ca_SRM915a_ values (i.e. using NIST SRM 915a as reference standard) for ease of comparison across studies. All Ca isotope compositions presented in the following text are δ^44/42^Ca_SRM915a_ values (noted δ^44/42^Ca for convenience as in previous studies; e.g. Heuser *et al*., Shroff *et al*., and Tanaka *et al*.^10^,^14^,^24^).

No Ca isotope reference material with a matrix similar to urine or blood exists, thus we used International Association for the Physical Sciences of the Oceans (IAPSO) seawater instead.^25^ The mean δ^44/42^Ca is 0.91‰ ± 0.02‰ [1 standard deviation (1 SD); *n* = 11], within error of the value of 0.94‰ ± 0.12‰ reported in Tacail*et al*.^25^ Precision was assessed by processing several aliquots of the seawater standard and yielded an uncertainty of 0.025‰ (1 SD; *n* = 11). The mean total procedure blank is 31 ng ± 21 ng of Ca (1 SD; *n* = 8), ∼0.1% of the amount of Ca processed for samples and affecting δ^44/42^Ca values by only ∼0.0002‰, well within the analytical uncertainty.

For statistical analysis, continuous variables are expressed as mean (standard deviation) or median (interquartile range) as per distribution. Categorical variables are expressed as number (percentage). Comparisons between groups, according to CKD status, were conducted using one-way ANOVA, χ^2^ and Kruskal–Wallis test as appropriate. Pearson's correlation test was used to assess association between the Ca isotope ratio of urine or serum, creatinine and other variables (creatinine and FGF-23 were log-transformed for data to be normally distributed). To determine which baseline variables were independently associated with the Ca isotope ratio of urine or serum, we performed linear regression analyses. The stronger determinants for serum Ca isotopes were selected by stepwise backward multivariable linear regression analysis. For inclusion, *P*-values were set at <0.2. Models were compared with adjusted *R*^2^ and the model with the largest value selected. We did not include CKD status in the multivariable linear regression analysis due to collinearity with creatinine. Analysis was conducted in SPSS (version 25) and R, and a *P*-value <0.05 was considered significant.^26^ Groups were not age matched, and age correction was not possible due to sample size, but the effect of age, as well as dietary and supplemental Ca, on interpretations are discussed in the following text.

The sensitivity and specificity for using calcium isotope ratios as a method of detecting vascular calcification were examined using the receiver operating characteristic (ROC) curve analysis for evaluating diagnostic tests and predictive models.^27^ ROC curve analysis was performed using R package, pROC.^28^ The reference method for detecting vascular calcification was an ABI ≤0.9 or ≥1.3 for PAD (suggesting intimal vascular calcification) and ba-PWV for arteriosclerosis (suggesting medial artery calcification). For arteriosclerosis, it was determined to be present if ba-PWV was ≥1800 cm/s or if ba-PWV was >0.16 × age^2^^ ^− 4.40 × age + 977.52 cm/s for female subjects or 0.20 × age^2^^ ^− 12.13 × age + 1341.34 cm/s for male subjects (where age is in years).^18^ An area under the curve (AUC) of 0.9–1 indicates an excellent diagnostic test; 0.8–0.89 a very good diagnostic test; 0.70–0.79 a good diagnostic test; 0.6–0.7 a sufficient diagnostic test; 0.5–0.6 a poor test; and <0.5 not useful test.^29^

## Results

### Clinical data

The median age of all participants was 60 [43–72] years, with participants in the control group being significantly younger (41 [29–56] years) than all other groups (*P* < 0.01). Overall, the majority of participants were male (*N* = 45, 58%). In the control group, the majority of participants were female (*N* = 16, 56%), whereas those in the other three groups were predominantly male (*P* < 0.01).

Body mass index (BMI) is not significantly different between groups (*P* = 0.10), nor is dietary Ca (*P* = 0.98; note for dietary Ca data were available for the mild–moderate CKD group). Two subjects in the control group took Ca medication (7% of that group), 6 in the mild–moderate CKD group (67%), 6 in the transplant group (35%), and 16 in the dialysis group (76%). As expected, participants undertaking dialysis show levels of PO_4_^3^^−^, PTH, ALP, creatinine, and FGF23 significantly higher than those in other groups (*P* < 0.01, Table 1). Additionally, they show reduced vitamin D levels compared to other groups (*P* < 0.01, Table 1). Fourteen (18%) participants exhibit an ABI of ≤0.9 indicating the presence of PAD (intimal calcification), but with no significant differences in proportions of participants with ABI ≤ 0.9 across all three CKD groups (*P* = 0.54, Table 1). For participants with an ABI > 0.9, the control group shows the highest proportion of participants (*N* = 23, 49%) (*P* < 0.01, Table 1).

**Table 1.** Demographics and biochemical data.

Characteristic	All	Control	Mild–moderate	Dialysis	Transplant	*P*-value
*n*	78	28	9	22	19	
Age (years)	59 [43–71]	38 [28–54] ^#^	70 [66–72]	71 [67–74]	58 [50–70]	<0.01*
Male	45 (58)	12 (44) ^#^	5 (56)	13 (59)	15 (79) ^#^	0.09
SBP (mmHg)	139 [122–159]	129 [118–141]	152 [145–179]	145 [140–166]	144 [138–161]	<0.01*
BMI (kg/m^2^)	28.0 [24.5–30.8]	26.0 [23.5–27.2]	29.8 [23.5–32.6]	29.1 [24.0–38.1]	28.7 [27.4–31.9]	0.102
eGFR (mL/min/1.73m^2^)		86 (7.2) ^#^	47 (24)	6.7 (2.0) ^#^	57 (21)	
PO_4_^3^^−^ (mmol/L)		1.13 (0.16)	1.12 (0.29)	1.51 (0.51) ^#^	1.03 (0.2)	<0.01*
Albumin (g/L)		40.8 (2.7)	39 (4.2)	32.6 (3.0) ^#^	41 (11)	<0.01*
PTH (pmol/L)		4.1 (3–5)	5 (4.3–8.5)	23 (4.1–54.6) ^ac^	7.8 (5.5–13.2) ^ac^	<0.01*
ALP (U/L)		69.5 (58–82.8)	82.5 (73.8–99)	120 (66–188) ^a^	73 (63.5–80) ^a^	<0.01*
Creatinine (μmol/L)		75 (65–81) ^#^	110 (87–151)	666 (519–739) ^#^	115 (93–149)	<0.01*
FGF23 (ng/L)		1.65 (1.55–1.76) ^#^	1.98 (1.79–2.07)	3.29 (3.14–4.19) ^#^	2.02 (1.87–2.21)	<0.01*
Vitamin D (1,25OH) (nmol/L)		120 (33.8)	112.6 (30.8)	44.24 (25.3) ^#^	109 (42.5)	<0.01*
PAD (ABI ≤ 0.9) ^i^		3 (4.9)	2 (3.3)	5 (8.2)	4 (6.6)	0.54
No PAD (ABI > 0.9) ^i^		23 (37.7)	5 (8.2)	8 (13.1) ^ac^	11 (18.0) ^a^	<0.01*
ba-PWV (cm/s) ^h^		1227 (229)	1694 (264)	2050 (713) ^a^	1585 (318) ^a^	<0.01*
Arteriosclerosis ^h^		3 (6.8)	3 (42.9)	6 (75) ^a^	9 (69.23) ^a^	<0.01*
Urine Ca (μg/g)		119 (86)	30 (38)	29 (20)	31 (22)	
Urine δ^44/42^Ca (‰)		0.54 (0.19)	0.48 (0.15)	0.69 (0.39)	0.92 (0.42)	
Serum Ca (μg/g)		57 (14)	n/a	59.6 (8.4)	60.9 (n/a)	
Serum δ^44/42^Ca (‰) ^g^		−0.70 (0.16)	−0.60 (0.14)	0.14 (0.24) ^ac^	−0.47 (0.16) ^a^	<0.01*

Continuous data expressed as mean (standard deviation) or median [interquartile range] as per distribution. Analysis by ANOVA or Kruskal–Wallis as per distribution. Categorical data expressed as number (percentage) and analysis with χ^2^. #: values are statistically significant from all others; ab: values are statistically significant from each other; *: values are statistically significant; a: values are statistically significant from control group; c: values are statistically significant from mild–moderate group; x: values are statistically significant between gender. FGF23: values reported are log10-transformed. i: *N* = 61; h: *N* = 51; g: *N* = 53.

The control group has significantly lower ba-PWV (1227 cm/s ± 229 cm/s; 1 SD) when compared to both the transplant (1586 cm/s ± 318 cm/s; 1 SD) and dialysis (2120 cm/s ± 790 cm/s; 1 SD) groups (*P* < 0.01), however, not when compared to the mild–moderate CKD group (*P* = 0.08, Table 2). Furthermore, the control group also has a lower prevalence of arteriosclerosis (medial calcification) as determined by ba-PWV (*N* = 3, 5.9%) (*P* < 0.01) than the dialysis and transplant group, however, not when compared to the mild–moderate CKD group (*P* = 0.08).

**Table 2.** Correlation of serum Ca isotope composition with covariables.

	Pearson correlation coefficient	*P*-value
Age (years)	0.50	<0.001
log(creatinine) (μmol/L)	0.83	<0.001
BMI (kg/m^2^)	0.09	0.53
SBP (mmHg)	0.25	0.08
ABI	0.17	0.30
ba-PWV (cm/s)	0.55	<0.001
Ca (mmol/L)	0.18	0.19
PO_4_^3^^−^ (mmol/L)	0.36	0.009
Albumin (g/L)	−0.72	<0.001
PTH (pmol/L)	0.26	0.06
ALP (U/L)	0.25	0.07
Vitamin D (1,25OH) (nmol/L)	−0.63	<0.001
log(FGF23) (ng/L)	0.78	<0.001

### Urine calcium concentration and isotope composition

**Fig. 1:**
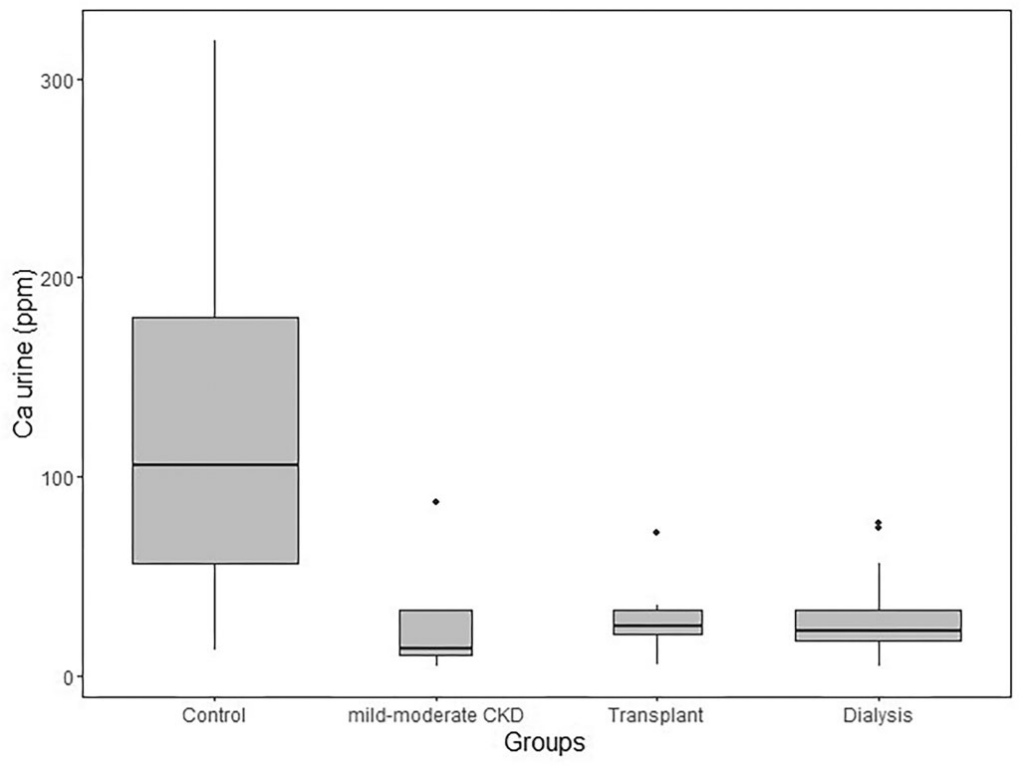
Boxplot of urine calcium concentrations (determined by ICP–MS) across the four groups. All figures were drawn using R package ggplot2.^37^

**Fig. 2:**
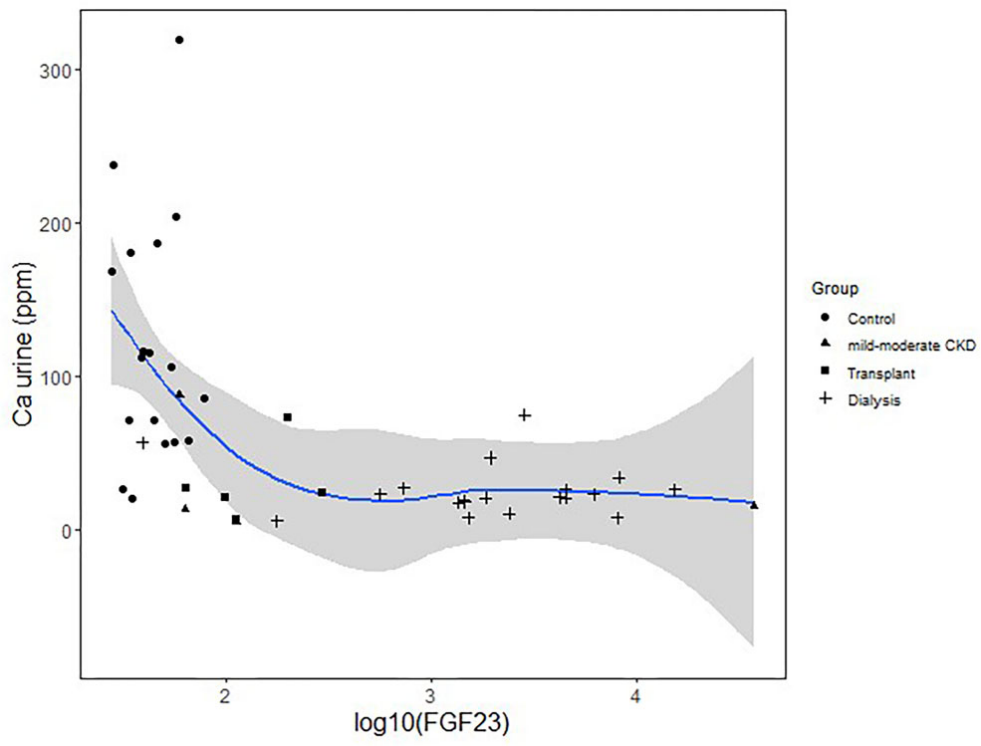
Urine Ca concentration (in μg/g) as a function of FGF23. The curve shows a local polynomial regression and the gray area represents the confidence interval at 0.9 level on the regression. Regressions and their confidence intervals were drawn in this and following figures using R package ggplot2^37^ with a span value (degree of smoothing) of 0.75 for polynomial regressions.

The mean urine Ca concentration (determined by ICP–MS) is 66 μg/g ± 72 μg/g. The mean urine Ca concentration of the control group (119 μg/g ± 86 μg/g) is significantly higher than that of each other group (*P* < 0.01 between control and mild–moderate CKD groups; *P* < 0.001 between control and dialysis or transplant groups; Fig. 1). There is a strong correlation (*P* < 0.001) between urine Ca and log-transformed creatinine (*R*^2^ = 0.21), log-transformed FGF23 (*R*^2^ = 0.26; Fig. 2), eGFR (*R*^2^ = 0.26), age (*R*^2^ = 0.19), and albumin (*R*^2^ = 0.18), and to a lesser extent with log-transformed PTH (*R*^2^ = 0.18, *P* = 0.002). The mean Ca isotope composition (δ^44/42^Ca) of urine is 0.61‰ ± 0.30‰ and shows no relationship with the CKD groups (not shown). We found poor correlations between urine δ^44/42^Ca and serum creatinine (*R*^2^ = 0.2, *P* = 0.3) and serum δ^44/42^Ca (*r* = 0.4, *P* = 0.06), and no correlations with other parameters.

### Serum ca isotope composition

**Fig. 3:**
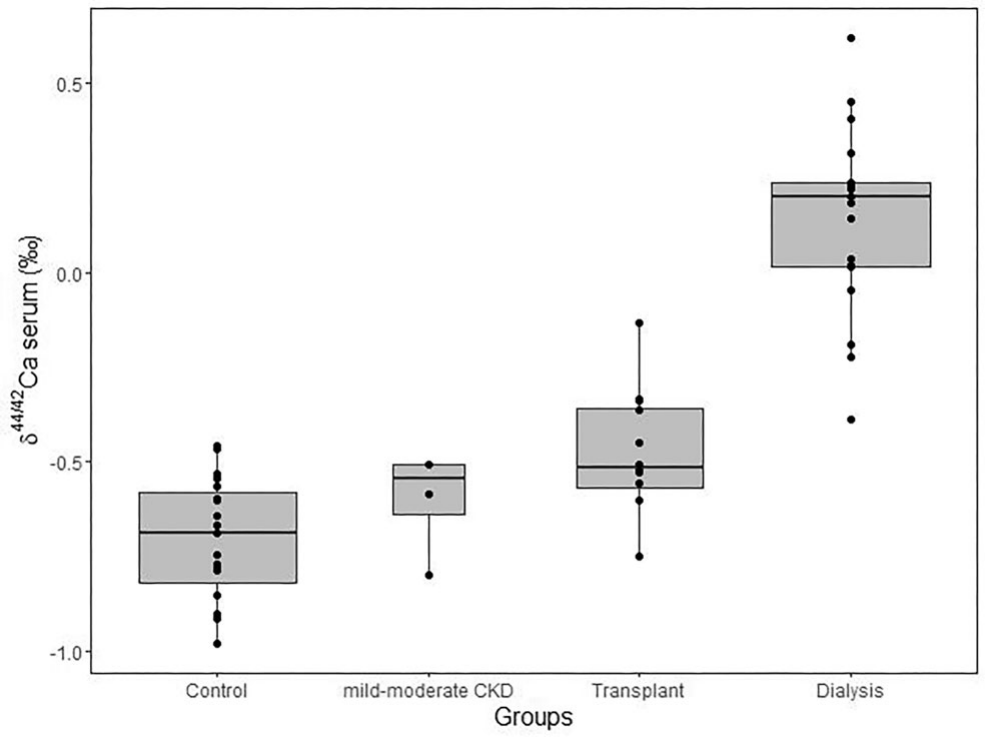
Boxplot of serum δ^44/42^Ca values across the four groups.

The mean δ^44/42^Ca of serum is −0.34‰ ± 0.42‰ (1 SD) with a significant difference across the four groups (*P* < 0.01). The mean δ^44/42^Ca of serum increases from controls (−0.70‰ ± 0.16‰; 1 SD, *N* = 10), to mild–moderate CKD (−0.60‰ ± 0.14‰; 1 SD, *N* = 4), to transplant (−0.47‰ ± 0.16‰; 1 SD, *N* = 12), and is highest in the dialysis group (0.14‰ ± 0.24‰; 1 SD, *N* = 19) (Fig. 3). There is no significant difference between the Ca isotope composition of serum in controls and that in the mild–moderate CKD group (*P* > 0.05). Each group was sub-sampled to yield a similar mean age, and the mean δ^44/42^Ca of each age-matched group was calculated. The age-matched control group has a mean δ^44/42^Ca of −0.77‰ ± 0.11‰ (1 SD, *N* = 9), not significantly different from that of the whole control group (*P* = 0.329). Similarly, the age-matched dialysis group has a mean δ^44/42^Ca of 0.17‰ ± 0.22‰ (1 SD, *N* = 8), also not significantly different for that of the whole dialysis group (*P* = 0.794).

**Fig. 4:**
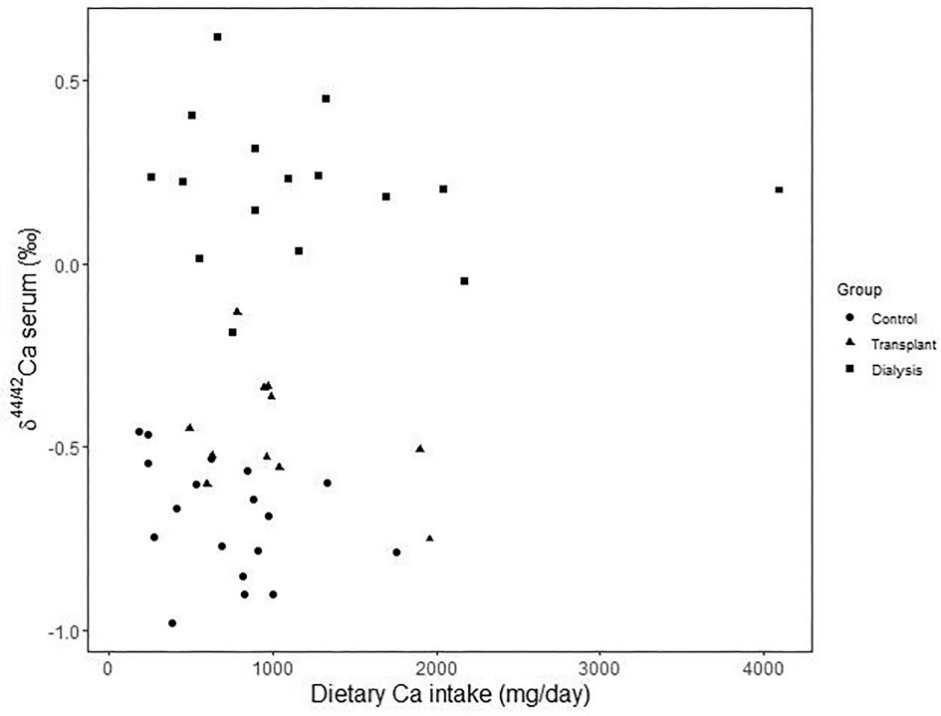
Serum δ^44/42^Ca (in ‰) as a function of dietary Ca intake (in mg), for three groups where both data were available. The lack of relationship suggests that dietary Ca may not have a major influence of serum Ca isotopes in our cohort.

**Fig. 5:**
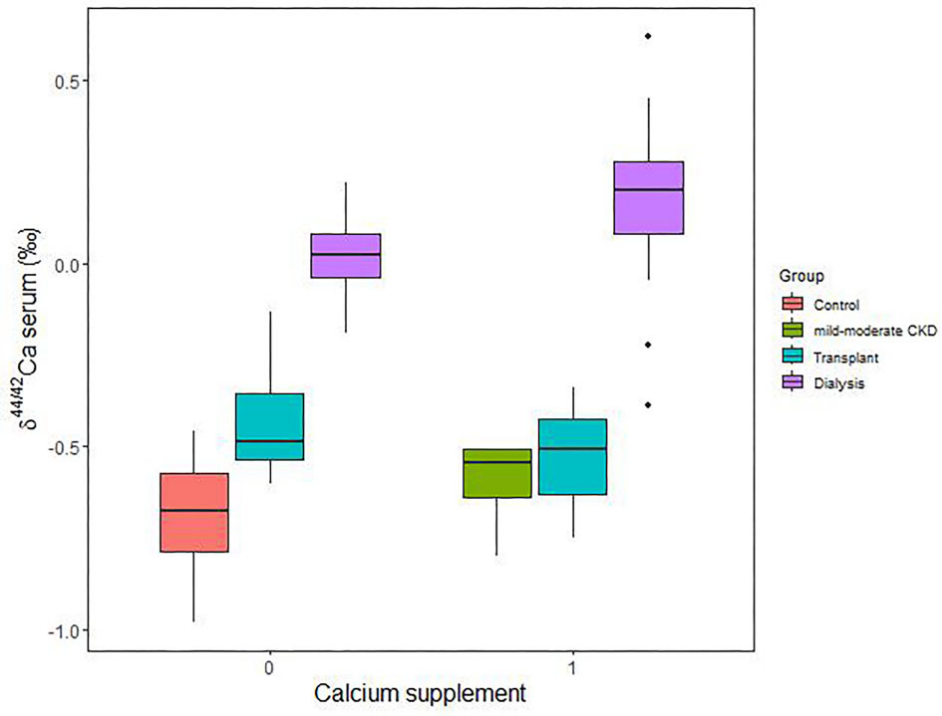
Serum δ^44/42^Ca (in ‰) as a function of whether calcium supplement/medication was taken. On the *x*-axis, “0” indicates no Ca supplement/medication taken, while “1” indicates Ca supplement/medication was taken. Note there was no serum δ^44/42^Ca data for the control subjects taking Ca supplement (*N* = 2), nor for the mild–moderate CKD subjects not taking Ca supplement (*N* = 3). The similarity in Ca isotope values for each transplant or dialysis, whether Ca supplement/medication was taken or not, suggests that Ca supplement/medication does not have a major influence on serum δ^44/42^Ca values.

There is no relationship between serum δ^44/42^Ca and dietary Ca intake (*P* = 0.156; Fig. 4). There are also no significant differences between the mean serum δ^44/42^Ca of transplant subjects taking Ca supplement, and that of those not taking any Ca supplement (*P* = 0.518). Similarly, there is no significant differences between the mean serum δ^44/42^Ca of dialysis subjects taking Ca medication as a phosphate binder, and that of those not taking any Ca medication (*P* = 0.197; Fig. 5).

**Fig. 6:**
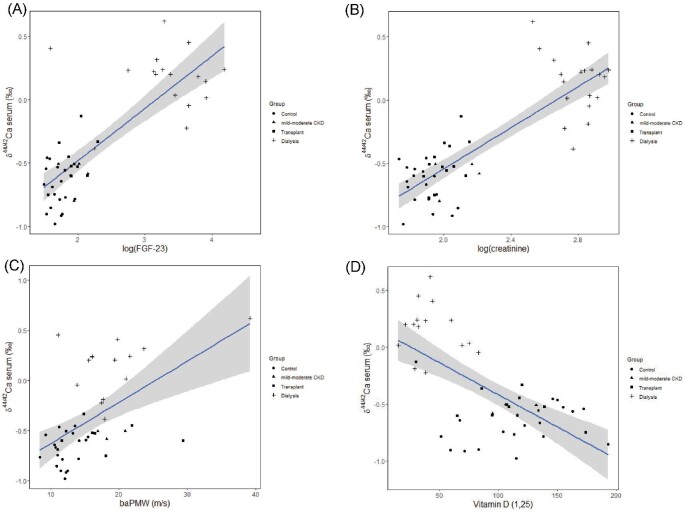
Serum Ca isotope composition as a function of (a) log-transformed FGF23; (b) log-transformed creatinine; (c) pulse wave velocity; and (d) vitamin D (1,25). The lines show linear regressions and gray areas represent the confidence interval at 0.9 level.

There are strong correlations (*P* < 0.001) between serum δ^44/42^Ca and log-transformed creatinine, log-transformed FGF23, PWV, vitamin D (Fig. 6), age, eGFR, and albumin (not shown). Serum δ^44/42^Ca was also significantly correlated with phosphate, ALP and PTH (Table 2). Positive correlation between serum δ^44/42^Ca and ALP is in agreement with the recent study of CKD children^14^; however, they also observed positive association between serum δ^44/42^Ca and vitamin D, and no correlation or a negative correlation between serum δ^44/42^Ca and PTH; however, here we observe a negative correlation between serum δ^44/42^Ca and vitamin D (Fig. 6), and positive between serum δ^44/42^Ca and PTH (not shown). In multivariate regression analysis, the main covariables strongly associated with serum δ^44/42^Ca are creatinine (*P* < 0.001) and PWV (*P* < 0.001) (Table 3).

**Fig. 7:**
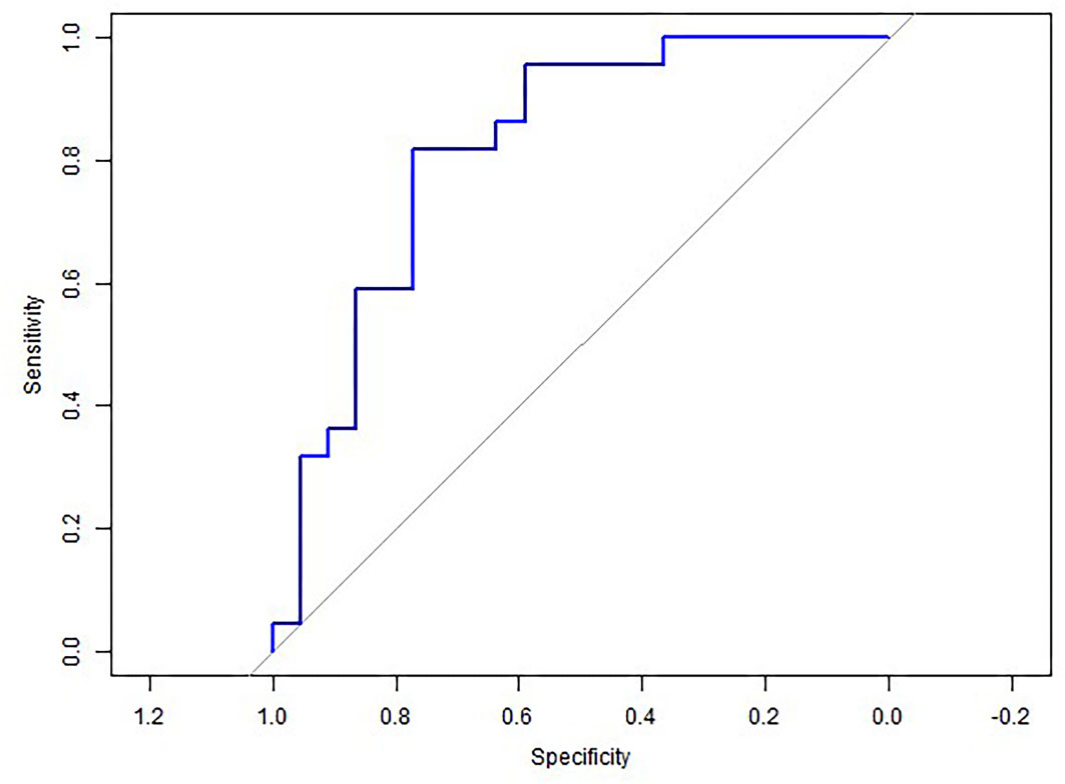
ROC curve for Ca isotopes in serum as medial calcification predictor.

**Table 3.** Association of serum Ca isotope composition with covariables.

	Linear regression		Backward linear regression	
	Beta (95% CI)	*P*-value	Beta (95% CI)	*P*-value
Age (years)	0.01	<0.01	−0.004	0.08
Female	0.07	0.6		
Group				
Control	Ref	Ref		
CKD	0.11	0.330		
Transplant	0.23	0.002		
Dialysis	0.84	<0.001		
Log(creatinine) (μmol/L)	0.82	<0.001	0.77	<0.001
BMI (kg/m^2^)	0.006	0.53		
SBP (mmHg)	0.004	0.085	−0.002	0.2
ABI	0.39	0.29		
ba-PWV (cm/s)	0.04	<0.001	0.025	0.001
Ca (mmol/L)	0.18	0.19		
PO_4_^3^^−^ (mmol/L)	0.37	0.009		
Albumin (g/L)	−0.06	<0.001	−0.02	0.02
PTH (pmol/L)	0.002	0.064	−0.002	0.04
ALP (U/L)	0.001	0.06		
Vitamin D (1,25OH) (nmol/L)	−0.006	<0.001		
log(FGF23) (ng/L)	0.41	<0.001		

Serum Ca isotope composition shows a strong association with medial artery calcification: ROC curve analysis shows that a serum δ^44/42^Ca value greater than −0.53‰ predicts presence of medial artery calcification with a sensitivity of 81.8% and specificity of 77.3%, with an AUC of 0.818 (Fig. 7). This diagnostic tool performs better than ba-PWV (sensitivity: 64.5%, specificity: 65.6% and AUC of 0.662, for a cut-off point at 1564 cm/s^30^).

## Discussion

Measurement of Ca isotopes in urine has been previously used to investigate and quantify changes in bone mineral balance,^9-14^,^16^,^23^,^31^ or in serum as a biomarker for multiple myeloma disease.^32^ Here, the Ca isotope composition of urine shows no systematic changes with CKD progression nor with medial or intimal calcification. This could be because, for logistical reasons, we collected spot urine samples instead of 24 hr urine. Although in our study, the conditions for urine collection were uniform across the cohort (in term of fasting and time of collection during the day), because urine composition is variable across the day, not being able to pool 24 hr urine could have resulted in blurring any association between urine δ^44/42^Ca and other biomarkers.

Tanaka *et al*.^24^ applied Ca isotopes in serum and bone to investigate bone mineral balance in CKD and diabetic rats. They found that serum Ca isotopes in rats were positively correlated with bone mineral density (measured on the right femoral bone using the DEXA method). Shroff *et al*.^14^ measured Ca isotopes in blood, urine and feces of a cohort of children affected with CKD, and children receiving dialysis therapy. Serum δ^44/42^Ca values in children with CKD and in children receiving dialysis were much lower than those of controls, interpreted as a loss of bone mineral content. Here, in our adult cohort, we observe the opposite, where subjects receiving dialysis display higher serum δ^44/42^Ca values than controls (and transplant subjects and subjects with mild–moderate CKD). The difference between the two studies could perhaps be explained as (i) in children, bone formation actively takes place, but not in adults; thus the study in children highlights the effect of bone loss in CKD subjects, and/or (ii) unlike adults, children with CKD experience vascular calcification to a much lesser extent than their adult counterparts; thus it is possible that the increase in serum Ca isotopes as a result of vascular calcification is minimal in children with CKD. While we note that the different groups are not age matched, since the median age of the dialysis group is greater than that of controls, and because bone resorption increases with age thus decreasing serum δ^44/42^Ca values,^11^,^12^ if we were able to correct each group for difference in bone mineral balance, the difference in serum δ^44/42^Ca values between the control and dialysis group could be even greater.

The Ca isotope composition of blood tracks changes in calcium mineral balance in the body, e.g. increasing as a result of bone mineralization.^11^ Similarly, the formation of Ca deposits in blood vessels could explain the observed increased serum δ^44/42^Ca in subjects showing signs of medial calcification (as defined by a ba-PWV value exceeding the threshold defined by age and sex^18^). The sensitivity of serum Ca isotopes to vascular calcification is surprisingly high, since arteriosclerosis would represent a small variation in Ca balance compared to bone mineralization.

Vascular calcification is common in CKD and is one of the strongest predictors of cardiovascular events and mortality.^33^ Its presence contributes to hypertension, increase PWV and left ventricular hypertrophy which all contribute to cardiovascular risk. While many risk factors of vascular calcification are themselves present in patients with CKD, such as older age, diabetes, hypertension, and smoking, it remains more common in the CKD population compared to a similarly aged population. The strong association between vascular calcification and risk of adverse events has generated a strong interest in interventions that can prevent progression or regress these lesions. However, they have been met with limited success. Vascular calcification is not due to a single entity but is rather a common endpoint to multiple pathological processes present in CKD such as hyperphosphatemia, hyperparathyroidism, vitamin D deficiency, hypertension, and disrupted calcium balance. A single drug intervention may therefore yield limited response and to date there is insufficient evidence and conflicting data that any intervention mitigates the risk of vascular calcification.^34^ Another challenge in targeting vascular calcification in CKD is the tools presently available for diagnosis. Imaging such as computed tomography and PWV are all able to diagnose vascular calcification in CKD and determine risk of cardiovascular events and mortality.^35^,^36^ However, they can only diagnose vascular calcification after permanent irreversible changes have set in, making interventions difficult to implement. A need to identify a marker for vascular calcification in the early stages, before clinical detection using traditional tools has occurred, is therefore needed. The physiological processes that disrupt Ca homeostasis occur early in CKD, before vascular calcification sets in. We have shown that serum Ca isotope measurements can detect vascular calcification, especially medial artery calcification in patients with CKD, therefore identifying it as a potential marker.

The need for a biomarker that can detect vascular calcification is not limited to being able to identify the disorder prior to irreversible changes occurring. It should also be easily measured for use as a screening population tool. Such a biomarker has the potential to allow for intervention, such as life-style changes or medical therapeutics, which may reverse or prevent the progression of vascular calcification early in the process. It can also be utilized as a tool to screen a large segment of the population who are at risk, such as those with CKD. Our study shows that serum Ca isotopes have the potential to fill this role since it utilizes a blood sample for detection of vascular calcification. However, the main disadvantage is that this method is currently only available in specialized laboratories.

Our main limitations are the small sample size and the cohort design with no prospective component. We recruited limited participants with early CKD compared to controls and KF. Since our study is a cohort analysis, we were unable to demonstrate causality with association in this cohort analysis. Finally, this study was only preformed in a single institution so the results will need to be verified in other settings and countries. Differing dietary practices could notably affect the baseline Ca isotope composition of serum and therefore potentially dim/reduce the sensitivity of serum Ca isotopes to vascular calcification. Culturally related dietary habits primarily linked to dairy products intake contribute interindividual variability (e.g. Tacail *et al*.^22^). Interestingly, for each group from the same Australian cohort, our results show a distribution of δ^44/42^Ca values with a typical spread (serum δ^44/42^Ca IQR ∼ 0.25‰) that is comparable to what has been reported in low and high dairy consuming populations (bone δ^44/42^Ca IQR = 0.2 and 0.3‰, respectively^22^) or in the control and osteoporotic women population from a previous clinical trial (urine δ^44/42^Ca IQR ∼ 0.2‰^11^). This suggests that the characterization of population baselines for groups with documented and homogeneous dietary habits would allow developing serum Ca isotopes as biomarkers for vascular calcification, in a similar way urine δ^44/42^Ca is used to track osteoporosis in women (e.g. Eisenhauer *et al*.^11^). Furthermore, at the scale of a cohort of individuals from a given culturally homogeneous group (women from Northern Germany), serum and urine δ^44/42^Ca values were shown to be only moderately influenced by intake of Ca from milk while primarily influenced sensitive to osteoporosis.^11^. Here, we showed that there is no association between serum δ^44/42^Ca and dietary Ca (Fig. 4), suggesting that diet has little influence on serum Ca isotopes in our cohort.

## Conclusion

This study set out to examine the association between naturally occurring calcium isotopes in serum and urine and its association with vascular calcification in a CKD population. Our results show that Ca isotopes in serum display a strong association to markers of medial calcification in patients with CKD (but not Ca isotopes in spot urine samples). ROC curve analysis indicates that Ca isotopes in serum perform well as a tool to detect medial artery calcification (sensitivity: 81.8%, specificity: 77.3%, AUC: 0.818). Thus, in the future, medial artery calcification could perhaps be detected in patients with CKD from a small (≤1 mL) blood sample. Confirmation will be required by conducting studies of other cohorts in addition to a prospective study design.

## Supplementary Material

mfad009_Supplemental_File

## Data Availability

All data are incorporated into the article and its online supplementary material.

## References

[1] Collaboration GBDCKD , Global, Regional, and National Burden of Chronic Kidney Disease, 1990-2017: a Systematic Analysis for the Global Burden of Disease Study 2017, Lancet, 2020, 395 (10225), 709–733. 10.1016/S0140-6736(20)30045-3 32061315 PMC7049905

[2] Jankowski J., Floege J., Fliser D., Böhm M., Marx N., Cardiovascular Disease in Chronic Kidney Disease, Circulation, 2021, 143 (11), 1157–1172. 10.1161/CIRCULATIONAHA.120.050686 33720773 PMC7969169

[3] Palit S., Kendrick J., Vascular Calcification in Chronic Kidney Disease: Role of Disordered Mineral Metabolism, Curr. Pharm. Des., 2014, 20 (37), 5829–5833. 10.2174/1381612820666140212194926 24533939 PMC4130905

[4] Ruderman I., Holt S. G., Hewitson T. D., Smith E. R., Toussaint N. D., Current and Potential Therapeutic Strategies for the Management of Vascular Calcification in Patients with Chronic Kidney Disease Including Those on Dialysis, Semin. Dial., 2018, 31 (5), 487–499. 10.1111/sdi.12710 29733462

[5] Krishnasamy R., Pedagogos E., Should Nephrologists Consider Vascular Calcification Screening? Nephrology, 2017, 22 (S2), 31–33. 10.1111/nep.13019 28429546

[6] Toepfer E. T., Rott J., Bartosova M., Kolevica A., Machuca-Gayet I., Heuser A., Rabe M., Shroff R., Bacchetta J., Zarogiannis S. G., Eisenhauer A., Schmitt C. P., Calcium Isotope Fractionation by Osteoblasts and Osteoclasts, Across Endothelial and Epithelial Cell Barriers and with Binding to Proteins, Am. J. Physiol. Regul. Integr. Comp. Physiol., 2021, 321 (1), R29–R40. 10.1152/ajpregu.00334.2020 33978493

[7] Tacail T. , Physiologie isotopique du calcium chez les mammifères, Ecole Normale Supérieure de Lyon. Lyon, France: Université de Lyon, 2017, 344

[8] Hassler A., Martin J. E., Ferchaud S., Grivault D., Le Goff S., Albalat E., Hernandez J.-A., Tacail T., Balter V., Lactation and Gestation Controls on Calcium Isotopic Compositions in a Mammalian Model, Metallomics, 2021, 13 (6). doi: 10.1093/mtomcs/mfab019 33881548

[9] Morgan J. L., Skulan J. L., Gordon G. W., Romaniello S. J., Smith S. M., Anbar A. D., Rapidly Assessing Changes in Bone Mineral Balance Using Natural Stable Calcium Isotopes, Proc. Natl. Acad. Sci. USA, 2012, 109 (25), 9989–9994. 10.1073/pnas.1119587109 22652567 PMC3382538

[10] Heuser A., Frings-Meuthen P., Rittweger J., Galer S. J. G., Calcium Isotopes in Human Urine as a Diagnostic Tool for Bone Loss: Additional Evidence for Time Delays in Bone Response to Experimental Bed Rest, Front Physiol., 2019, 10, 12. 10.3389/fphys.2019.00012 30740058 PMC6355708

[11] Eisenhauer A., Müller M., Heuser A., Kolevica A., Glüer C. C., Both M., Laue C., Hehn U. v., Kloth S., Shroff R., Schrezenmeir J., Calcium Isotope Ratios in Blood and Urine: a New Biomarker for the Diagnosis of Osteoporosis, Bone Rep., 2019, 10, 100200. 10.1016/j.bonr.2019.100200 30997369 PMC6453776

[12] Shroff R., Fewtrell M., Heuser A., Kolevica A., Lalayiannis A., McAlister L., Silva S., Goodman N., Schmitt C. P., Biassoni L., Rahn A., Fischer D. C., Eisenhauer A., Naturally Occurring Stable Calcium Isotope Ratios in Body Compartments Provide a Novel Biomarker of Bone Mineral Balance in Children and Young Adults, J. Bone Miner. Res., 2021, 36 (1), 133–142. 10.1002/jbmr.4158 32786145

[13] Heuser A., Eisenhauer A., A Pilot Study on the Use of Natural Calcium Isotope (44Ca/40Ca) Fractionation in Urine as a Proxy for the Human Body Calcium Balance, Bone, 2010, 46 (4), 889–896 10.1016/j.bone.2009.11.037 20004263

[14] Shroff R., Lalayiannis A. D., Fewtrell M., Schmitt C. P., Bayazit A., Askiti V., Jankauskiene A., Bacchetta J., Silva S., Goodman N., McAlister L., Biassoni L., Crabtree N., Rahn A., Fischer D.-C., Heuser A., Kolevica A., Eisenhauer A., Naturally Occurring Stable Calcium Isotope Ratios Are a Novel Biomarker of Bone Calcium Balance in Chronic Kidney Disease, Kidney Int., 2022, 102 (3), 613–623. 10.1016/j.kint.2022.04.024 35644284

[15] Yamada S., Giachelli C. M., Vascular Calcification in CKD-MBD: Roles for Phosphate, FGF23, and Klotho, Bone, 2017, 100, 87–93. 10.1016/j.bone.2016.11.012 27847254 PMC5429216

[16] Channon M. B., Gordon G. W., Morgan J. L., Skulan J. L., Smith S. M., Anbar A. D., Using Natural, Stable Calcium Isotopes of Human Blood to Detect and Monitor Changes in Bone Mineral Balance, Bone, 2015, 77, 69–74. 10.1016/j.bone.2015.04.023 25900894

[17] Tomiyama H., Yamashina A., The Application of Brachial-Ankle Pulse Wave Velocity as a Clinical Tool for Cardiovascular Risk Assessment, Hypertension, 2012, 60 (5), e40; author reply e1 10.1161/HYPERTENSIONAHA.112.201806 22966011

[18] Chen S. C., Chang J.-M., Liu W.-C., Tsai Y.-C., Tsai J. C., Hsu P.-C., Lin T.-H., Lin M.-Y., Su H.-M., Hwang S.-J., Chen H. C., Brachial-Ankle Pulse Wave Velocity and Rate of Renal Function Decline and Mortality in Chronic Kidney Disease, Clin. J. Am. Soc. Nephrol., 2011, 6 (4), 724–732 10.2215/CJN.07700910 21454721 PMC3069362

[19] Romaniello S. J., Field M. P., Smith H. B., Gordon G. W., Kim M. H., Anbar A. D., Fully Automated Chromatographic Purification of Sr and Ca for Isotopic Analysis, J. Anal. At. Spectrom., 2015, 30 (9), 1906–1912. 10.1039/C5JA00205B

[20] Albarède F., Beard B., Analytical Methods for Non-Traditional Isotopes, Rev. Mineral. Geochem., 2004, 55 (1), 113–152 10.2138/gsrmg.55.1.113

[21] Tacail T., Albalat E., Télouk P., Balter V., A Simplified Protocol for Measurement of Ca Isotopes in Biological Samples, J. Anal. At. Spectrom., 2014, 29 (3), 529. 10.1039/c3ja50337b

[22] Tacail T., Martin J. E., Herrscher E., Albalat E., Verna C., Ramirez-Rozzi F., Clark G., Valentin F., Balter V., Quantifying the Evolution of Animal Dairy Intake in Humans Using Calcium Isotopes, Quat. Sci. Rev., 2021, 256, 106843. 10.1016/j.quascirev.2021.106843

[23] Morgan J. L., Gordon G. W., Arrua R. C., Skulan J. L., Anbar A. D., Bullen T. D., High-Precision Measurement of Variations in Calcium Isotope Ratios in Urine by Multiple Collector Inductively Coupled Plasma Mass Spectrometry, Anal. Chem., 2011, 83 (18), 6956–6962. 10.1021/ac200361t 21740001

[24] Tanaka Y. K., Yajima N., Higuchi Y., Yamato H., Hirata T., Calcium Isotope Signature: New Proxy for Net Change in Bone Volume for Chronic Kidney Disease and Diabetic Rats, Metallomics, 2017, 9 (12), 1745–1755. 10.1039/C7MT00255F 29115324

[25] Tacail T., Télouk P., Balter V., Precise Analysis of Calcium Stable Isotope Variations in Biological Apatites Using Laser Ablation MC-ICPMS, J. Anal. At. Spectrom., 2016, 31 (1), 152–162. 10.1039/C5JA00239G

[26] Fisher R A. , Statistical Methods for Research Workers. Edinburgh: Oliver and Boyd, 1926.

[27] Zou K. H., o Malley A. J., Mauri L., Receiver-Operating Characteristic Analysis for Evaluating Diagnostic Tests and Predictive Models, Circulation, 2007, 115 (5), 654–657. 10.1161/CIRCULATIONAHA.105.594929 17283280

[28] Robin X., Turck N., Hainard A., Tiberti N., Lisacek F., Sanchez J.-C., Müller M., pROC: an Open-Source Package for R and S+ to Analyze and Compare ROC Curves, BMC Bioinf., 2011, 12 (1), 77. 10.1186/1471-2105-12-77 PMC3068975 21414208

[29] Hanley J. A., McNeil B. J., The Meaning and Use of the Area under a Receiver Operating Characteristic (ROC) Curve, Radiology, 1982, 143 (1), 29–36. 10.1148/radiology.143.1.7063747 7063747

[30] Liu C. S., Li C.-I., Shih C.-M., Lin W.-Y., Lin C.-H., Lai S. W., Li T.-C., Lin C.-C., Arterial Stiffness Measured as Pulse Wave Velocity is Highly Correlated with Coronary Atherosclerosis in Asymptomatic Patients, J. Atheroscler. Thromb., 2011, 18 (8), 652–658 10.5551/jat.7021 21467807

[31] Skulan J., DePaolo D. J., Calcium Isotope Fractionation between Soft and Mineralized Tissues as a Monitor of Calcium Use in Vertebrates, Proc. Natl. Acad. Sci. USA, 1999, 96 (24), 13709–13713. 10.1073/pnas.96.24.13709 10570137 PMC24129

[32] Gordon G. W., Monge J., Channon M. B., Wu Q., Skulan J. L., Anbar A. D., Fonseca R., Predicting Multiple Myeloma Disease Activity by Analyzing Natural Calcium Isotopic Composition, Leukemia, 2014, 28 (10), 2112–2115. 10.1038/leu.2014.193 24919808

[33] Liu M., Li X. C., Lu L., Cao Y., Sun R. R., Chen S., Zhang P. Y., Cardiovascular Disease and Its Relationship with Chronic Kidney Disease, Eur. Rev. Med. Pharmacol. Sci., 2014, 18 (19), 2918–2926 25339487

[34] Xu C., Smith E., Tiong M., Ruderman I., Toussaint N., Interventions to Attenuate Vascular Calcification Progression in Chronic Kidney Disease: a Systematic Review of Clinical Trials, J. Am. Soc. Nephrol., 2022, 33 (5), 1011–1032. 10.1681/ASN.2021101327 35232774 PMC9063901

[35] Chen J., Budoff M. J., Reilly M. P., Yang W., Rosas S. E., Rahman M., Zhang X., Roy J. A., Lustigova E., Nessel L., Ford V., Raj D., Porter A. C., Soliman E. Z., Wright J. T. Jr, Wolf M., He J., Investigators C, Coronary Artery Calcification and Risk of Cardiovascular Disease and Death among Patients with Chronic Kidney Disease, JAMA Cardiol., 2017, 2 (6), 635–643. 10.1001/jamacardio.2017.0363 28329057 PMC5798875

[36] Krishnasamy R., Tan S. J., Hawley C. M., Johnson D. W., Stanton T., Lee K., Mudge D. W., Campbell S., Elder G. J., Toussaint N. D., Isbel N. M., Progression of Arterial Stiffness Is Associated with Changes in Bone Mineral Markers in Advanced CKD, BMC Nephrol., 2017, 18 (1), 281. 10.1186/s12882-017-0705-4 28870151 PMC5584006

[37] Wickham H. , ggplot2: Elegant Graphics for Data Analysis. Springer-Verlag New York, 2016.

